# Enteral nutrition–related hyperglycemia in critical illness: cascade conceptual model and implications for precision nursing—a systematic review

**DOI:** 10.3389/fnut.2026.1803501

**Published:** 2026-07-07

**Authors:** Jiani Qian, Quanquan He, Maoyun Miao, Weiwei Ni, Yulei Ma

**Affiliations:** 1 School of Nursing, Shandong Second Medical University, Weifang, China; 2 Department of Nursing, Affiliated Hospital of Shandong Second Medical University, Weifang, China; 3 Weifang Key Laboratory for Clinical Nursing Research, Department of Nursing, Affiliated Hospital of Shandong Second Medical University, Weifang, China

**Keywords:** blood glucose, critical illness, enteral nutrition, glucose monitoring, continuous, hyperglycemia, insulin, nursing care

## Abstract

**Background:**

Hyperglycemia is a common complication in critically ill patients receiving enteral nutrition (EN). This review synthesizes evidence on its mechanisms, epidemiology, clinical implications, and nursing management to support precision glycemic care.

**Methods:**

A systematic literature search identified 45 eligible studies, including randomized trials, retrospective studies, reviews, guidelines, and expert consensus statements. Given the lack of a universally accepted definition, enteral nutrition-related hyperglycemia was pragmatically defined as blood glucose ≥7.8 mmol/L occurring within 24−72 h after initiation of enteral nutrition.

**Results:**

Reported incidence varies widely, from approximately 30−47% in general ICU populations to 60−80% following cardiac surgery, reflecting differences in definitions, patient characteristics, timing, and nutritional protocols. Based on thematic integration of existing evidence, we propose a six-level conceptual cascade framework, linking metabolic vulnerability, stress-hormone activation, exogenous substrate load, enteroinsular axis dysfunction, inflammation-mediated insulin resistance, and microbiota dysbiosis. Corresponding nursing strategies include diabetes-specific formulas, optimized feeding regimens, continuous glucose monitoring, and individualized insulin protocols within multidisciplinary care. These strategies may improve glycemic control; however, evidence for clinical outcome benefits remains limited and heterogeneous.

**Conclusion:**

Further prospective studies are needed to validate the model and refine precision nursing strategies.

## 1 Introduction

Enteral Nutrition (EN) remains the preferred method of nutritional support in the intensive care unit (ICU) due to its physiological advantages, including preservation of gut integrity and reduction of bacterial translocation (1). However, its carbohydrate-rich formulation can aggravate hyperglycemia, particularly in patients with compromised insulin sensitivity. Among patients receiving EN, approximately one-third develop hyperglycemia, although the reported incidence varies depending on the definition of hyperglycemia, patient characteristics, and monitoring strategies (2, 3). The metabolic alterations triggered by sepsis, trauma, or major surgery stimulate a surge of counter-regulatory hormones that enhance hepatic glucose output and promote insulin resistance. Although this stress-driven rise in blood glucose may offer short-term benefits, sustained hyperglycemia is strongly linked to impaired immune responses, higher infection rates, delayed wound healing, and increased mortality (4). Observational studies consistently associate dysglycemia with prolonged mechanical ventilation, greater susceptibility to sepsis, and extended ICU and hospital stays (5, 6).

However, current research mainly focuses on isolated mechanisms or single risk factors of EN-related hyperglycemia, without a comprehensive framework integrating its multifactorial nature (4). In addition, prior studies have insufficiently addressed heterogeneity among patients in ICUs. This review therefore synthesizes current evidence on EN-related hyperglycemia, including its epidemiology, mechanisms, and corresponding nursing interventions. It focuses on adult patients in general ICUs while acknowledging differences across patient subgroups. Based on existing evidence, we propose a six-level cascade model integrating metabolic susceptibility, stress-hormone activation, exogenous substrate load, enteroinsular axis dysfunction, inflammation-related insulin resistance, and gut microbiota dysregulation. This model is a conceptual, theory-driven framework that has not yet been clinically validated. It may provide a foundation for developing precision nursing strategies, such as individualized formula selection, tailored feeding regimens, dynamic glucose monitoring, and structured insulin therapy within multidisciplinary care. By integrating evidence and recommendations from international nutrition and critical care guidelines (including ASPEN and other relevant societies) with mechanistic and clinical perspectives, this review aims to support individualized glycemic management (7).

## 2 Methods

### 2.1 Search strategy

A literature search was conducted across PubMed, Web of Science, Embase, the Cochrane Library, Scopus, and CINAHL. The search combined Medical Subject Headings (MeSH) and relevant keywords related to critical illness, hyperglycemia and stress hyperglycemia, enteral nutrition, and glycemic management. Only English-language studies published from database inception through November 2025 were considered. This timeframe allowed inclusion of evolving guidelines and recent practice updates while avoiding outdated evidence less applicable to contemporary ICU care.

### 2.2 Inclusion and exclusion criteria

Studies were eligible if they: (1) involved adult hospitalized patients receiving enteral nutrition; (2) examined hyperglycemia, glycemic variability, or glucose-related outcomes; and (3) used designs such as randomized controlled trials, observational studies, systematic reviews, meta-analyses, clinical guidelines, or expert consensus statements. Studies were excluded if they were duplicate reports, abstracts without full texts, commentary-type publications, non-English sources, or lacked relevant outcomes. These criteria were chosen to maintain methodological rigor and ensure the relevance of synthesized findings to nursing and clinical practice.

### 2.3 Literature screening and data extraction

**Figure 1:**
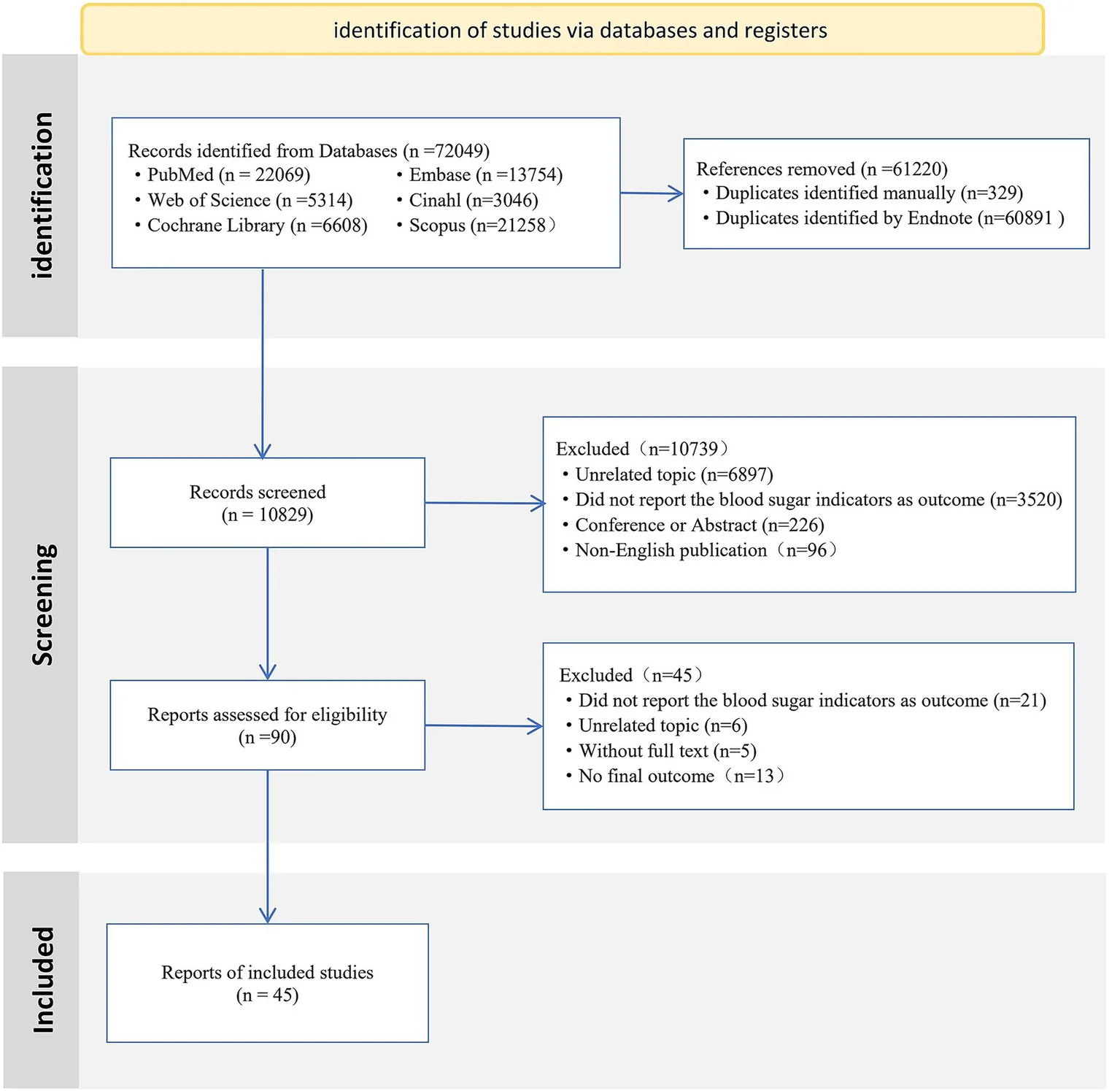
PRISMA flow diagram illustrating the literature search and selection process. This figure details the step-by-step inclusion and exclusion of studies, from 72,049 initial database records to the final set of 45 articles, highlighting reasons for exclusions at each stage to ensure methodological transparency.

Screening was conducted in two stages by two independent reviewers, with disagreements resolved by discussion or consultation with a third reviewer. A total of 45 studies were included in the final analysis (see Figure 1). The detailed literature search strategy, search results, and characteristics of the included studies are provided in Supplementary Table 1. Data were extracted using a structured form capturing publication year, first author, study design, and the main content of the article. For the purpose of data synthesis, and given the lack of a universally accepted definition, enteral nutrition-related hyperglycemia was pragmatically defined as blood glucose ≥7.8 mmol/L occurring within 24–72 h after initiation of enteral nutrition. Cross-checking was performed to ensure accuracy and consistency of data extraction.

### 2.4 Quality assessment and bias control

Study quality was assessed using the Cochrane Risk of Bias 2 tool for randomized trials and the Newcastle–Ottawa Scale for observational studies. Reviews and guidelines were appraised based on methodological rigor and recommendation quality. Overall, the included evidence was heterogeneous, with observational studies generally of moderate quality and randomized trials showing variable risk of bias. Assessments were used to aid interpretation rather than to exclude studies or weight findings.

### 2.5 Data synthesis and reporting standards

This systematic review followed a structured search strategy and transparent screening process in accordance with the PRISMA 2020 guidelines. Meta-analysis was not performed because the included studies differed substantially in design, patient populations, definitions of hyperglycemia, enteral nutrition protocols, and glycemic management strategies. Therefore, findings were synthesized using a narrative and thematic approach to identify recurring mechanistic pathways, clinical patterns, and implications for glycemic management in critically ill patients receiving enteral nutrition.

## 3 Results

### 3.1 Epidemiological characteristics of hyperglycemia related to enteral nutrition in critically ill patients

To delineate the clinical profile and implications of EN-related hyperglycemia in critically ill patients, Table 1 synthesizes key evidence across incidence, prognostic impacts, high-risk factors, and glycemic variability, drawing from diverse study designs.

**Table 1:** Key epidemiological characteristics of enteral nutrition–related hyperglycemia in critically ill patients.

Enteral nutrition-related hyperglycemia in critically ill: key epidemiology		
Domain	Key findings	Clinical notes
Incidence	1) Occurs in 30–47% of general ICU patients 2) Up to 60–80% following cardiac surgery	Hyperglycemia is particularly pronounced in non-diabetic patients exposed to acute stress.
Prognostic impact	1) Associated with higher mortality, increased infections, and prolonged ICU/hospital stay 2) Glycemic variability (GV) independently predicts adverse outcomes	Non-diabetic hyperglycemia often reflects severe illness and carries strong prognostic significance.
Major risk factors	1) Pre-existing diabetes or insulin resistance 2) Obesity and advanced age 3) High-carbohydrate EN formulas or rapid caloric escalation 4) Glucocorticoid and vasopressor use Sepsis, inflammation, and multi-organ dysfunction	Most risk factors converge on impaired insulin signaling and reduced metabolic flexibility.

This table summarizes the principal epidemiological features of hyperglycemia associated with enteral nutrition in adult ICU patients, including prevalence across clinical populations, prognostic implications and major risk factors. The table provides a concise overview to complement the detailed discussion of mechanisms and clinical considerations presented in the main text.

### 3.2 The six-level cascade pathophysiological model of hyperglycemia related to enteral nutrition

**Figure 2:**
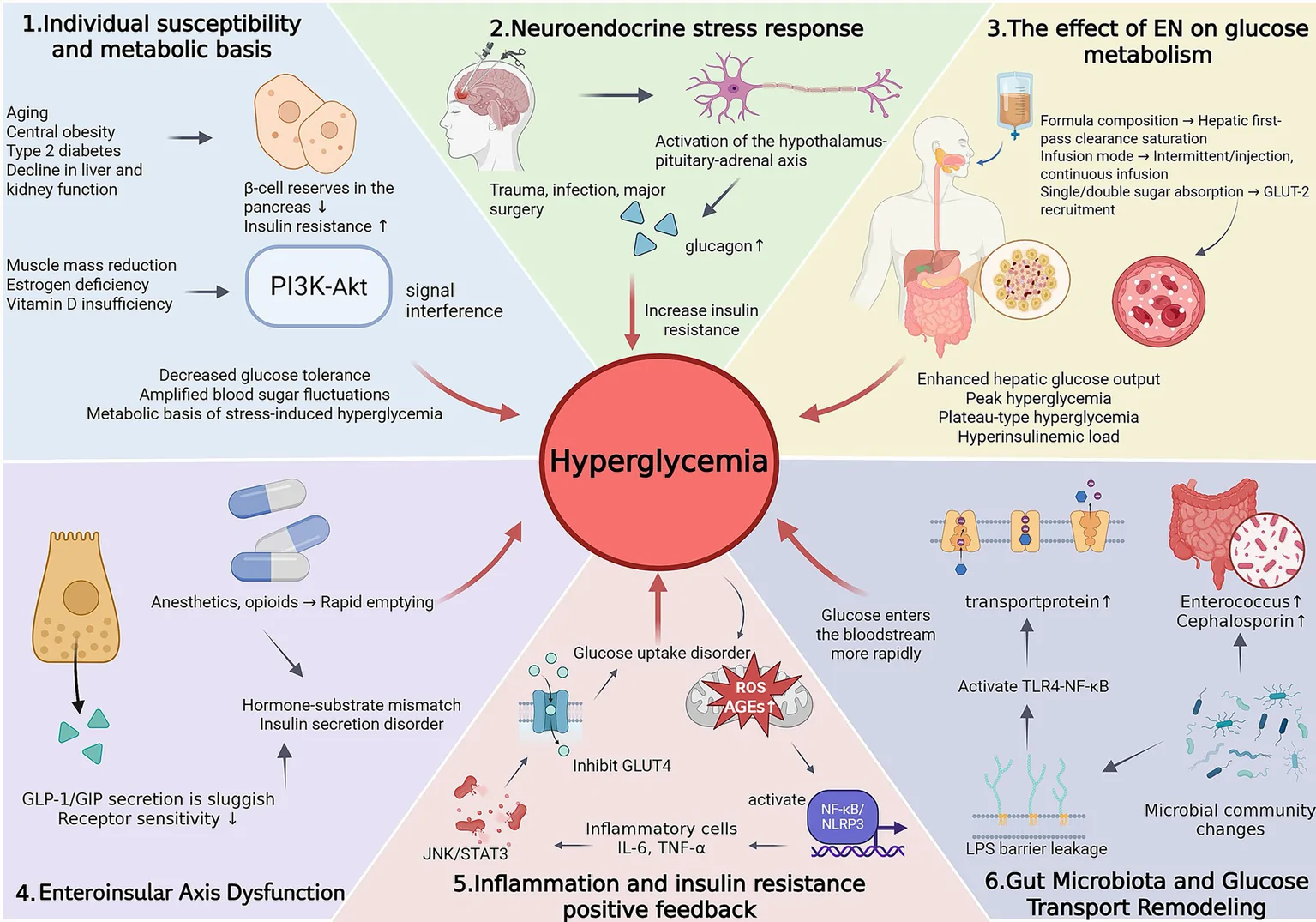
Schematic illustration of six-level cascade mechanisms underlying hyperglycemia in critically ill patients receiving enteral nutrition. This figure illustrates interconnected pathophysiological pathway. (1) Individual susceptibility and metabolic background: Aging, central obesity, type 2 diabetes, reduced *β*-cell reserve, muscle loss, estrogen/vitamin D deficiency, and PI3K–Akt disruption impair glucose tolerance and increase variability. (2) Neuroendocrine stress response: Trauma, infection, or surgery activates the hypothalamic–pituitary–adrenal axis, increasing glucagon and insulin resistance. (3) Enteral nutrition effects: Formula composition and feeding pattern, together with GLUT-2–mediated absorption, promote hepatic saturation, increased glucose output, and peak/plateau hyperglycemia. (4) Enteroinsular axis dysfunction: Delayed GLP-1/GIP secretion, receptor desensitization, and hormone–substrate mismatch impair insulin regulation. (5) Inflammation–insulin resistance loop: ROS/AGEs and JNK/STAT3, IL-6/TNF-α, NF-κB/NLRP3 pathways suppress GLUT4 and amplify inflammation. (6) Gut microbiota remodeling: Microbiota changes and TLR4–NF-κB activation upregulate glucose transporters, accelerating glucose entry. Arrows indicate directional interactions and amplification across levels, forming a cascade in which each level ultimately converges toward the common endpoint of hyperglycemia.

The development of enteral nutrition–related hyperglycemia in critically ill patients involves multiple interacting mechanisms, including metabolic vulnerability, stress hormone activation, exogenous glucose loading, enteroinsular axis dysfunction, inflammation-associated insulin resistance, and gut microbiota alterations. Although these mechanisms have been individually described in previous studies, they are generally reported as separate and fragmented pathways. Accordingly, they were organized into a six-level cascade framework according to their predominant mechanistic roles and temporal progression. This framework provides a structured representation of the dynamic and interrelated processes underlying enteral nutrition–related hyperglycemia. The six levels are defined by dominant pathophysiological drivers rather than strictly separated biological events, as several processes such as inflammation and insulin resistance may span multiple stages. Therefore, the boundaries between levels are conceptual and reflect functional predominance rather than absolute separation. As an integrative schematic, the model summarizes current evidence on the pathophysiology of enteral nutrition–related hyperglycemia (Figure 2).

#### 3.2.1 Initiating factors: host metabolic reserves and intrinsic susceptibility

The metabolic response to enteral nutrition is strongly influenced by a patient’s baseline physiological reserve. Advanced age, central obesity, type 2 diabetes, and impaired hepatic or renal function are associated with reduced β-cell responsiveness and increased insulin resistance, thereby limiting the capacity to handle exogenous glucose loads (8–11). These alterations create a metabolic milieu in which even modest increases in carbohydrate delivery can provoke disproportionate glycemic excursions. Additional factors such as sarcopenia, estrogen deficiency, and inadequate vitamin D status may further impair insulin signaling through disruptions in the PI3K–Akt pathway. Collectively, these features represent a vulnerable metabolic state that predispose patients to stress hyperglycemia during critical illness and enteral feeding. Identifying these risk features before initiating EN can therefore help anticipate glycemic instability and guide early intervention.

#### 3.2.2 Core driver: stress-mediated neuroendocrine and cytokine activation

Critical illness triggers profound neuroendocrine and inflammatory activation that exerts a dominant influence on glucose homeostasis. Trauma, infection, and major surgery stimulate both the hypothalamic–pituitary–adrenal axis and the sympathetic nervous system, resulting in marked increases in catecholamines, glucocorticoids, glucagon, and growth hormone (12–14). These hormones collectively accelerate hepatic glycogenolysis and gluconeogenesis, suppress endogenous insulin secretion, and intensify peripheral insulin resistance. Their concentrations often rise in proportion to illness severity; for example, catecholamine levels in sepsis may increase several-fold, contributing to the insulin refractory state frequently observed in the ICU (14). In addition to endogenous sympathetic activation, exogenous catecholamine exposure, particularly through vasopressor therapy commonly used in critically ill patients, may further exacerbate this hyperglycemic response (15). In parallel, proinflammatory cytokines amplify these metabolic disruptions by impairing insulin signaling at the receptor level. This stress-driven hormonal and inflammatory milieu promotes hyperglycemia, which is further exacerbated by enteral nutrient delivery.

#### 3.2.3 Metabolic challenge: enteral nutrition as an exogenous substrate load

Once enteral nutrition is initiated, exogenous nutrient delivery imposes an additional metabolic challenge. In standard formulas, carbohydrates account for more than 50% of total energy intake. Under physiological conditions, the liver buffers intestinal glucose influx through increased uptake and glycogen storage; however, this capacity may be insufficient under metabolic stress or dysfunction, leading to increased systemic glucose exposure and hyperglycemia (16). Feeding patterns also influence glycemic responses, as bolus administration may induce rapid glucose excursions due to faster gastric emptying, whereas continuous infusion is associated with lower variability but sustained mild hyperglycemia. Formulas rich in rapidly absorbable mono- and disaccharides may further amplify postprandial glycemic responses through enhanced intestinal glucose transport. These interactions highlight the need for individualized nutritional strategies in glycemic management in critically ill patients.

#### 3.2.4 Endocrine dysregulation: enteroinsular axis dysfunction

Critical illness can impair the enteroinsular axis, disrupting the normal coordination between nutrient delivery, incretin signaling, and pancreatic insulin secretion. Inflammatory responses and ischemia–reperfusion injury blunt the release and effectiveness of key hormones such as GLP-1 and GIP, while sedatives and opioids commonly used in the ICU delay gastric emptying (17). The resulting mismatch between nutrient entry and hormonal signaling may lead to exaggerated glucose rises, a pattern often seen when delayed gastric emptying suddenly transitions into rapid small-bowel nutrient exposure. Small physiological studies and early clinical trials suggest that incretin-based therapies, particularly GLP-1 receptor agonists, may attenuate nutrient-stimulated hyperglycemia and reduce insulin requirements (18); however, current evidence remains limited and is largely derived from small or early-phase studies. Therefore, their clinical applicability in critically ill populations remains uncertain.

#### 3.2.5 Vicious cycle: amplification of hyperglycemia and insulin resistance

As hyperglycemia becomes established, it contributes to a self-perpetuating cycle of metabolic deterioration. Elevated glucose levels increase oxidative stress and promote formation of advanced glycation end-products, activating inflammatory pathways such as NF-κB and the NLRP3 inflammasome. These responses deepen insulin resistance by inhibiting insulin receptor substrate activity and reducing GLUT-4 translocation (19). In this context, even consistent EN delivery can produce unpredictable glycemic swings, reflected clinically in increased glycemic variability—a parameter strongly associated with mortality in ICU cohorts (20). This bidirectional amplification between hyperglycemia and inflammation underscores the need for management strategies that address both metabolic and inflammatory drivers.

#### 3.2.6 System reengineering: microbiota dysbiosis and altered glucose absorption

The final stage of the cascade model involves gut microbiota dysbiosis, which is common in critically ill patients and is associated with disease severity, antibiotic exposure, and enteral nutrition. This state is characterized by reduced microbial diversity, increased abundance of opportunistic pathogens, and impaired intestinal barrier integrity, which facilitates translocation of microbial products and activation of inflammatory pathways such as TLR4 signaling (21). Intestinal glucose absorption is primarily mediated by SGLT1 and GLUT-2, whose activity is dynamically regulated by luminal glucose availability and inflammatory status (22). Microbiota-derived metabolites may further influence glucose metabolism through effects on inflammation, barrier function, and gut endocrine signaling. Although microbiota-targeted interventions may represent a potential strategy to improve metabolic and inflammatory control, current evidence in critically ill populations remains limited.

### 3.3 Precision management based on the cascade model: from pathophysiological targets to clinical interventions

In addition to endogenous pathophysiological processes, multiple exogenous and clinical factors, including pharmacological treatments, insulin protocols, caloric load, organ dysfunction, feeding interruptions, and ICU practices, interact across the cascade as cross-cutting modifiers that influence its progression. Within this framework, nursing functions as a key operational interface linking metabolic monitoring with intervention delivery. Accordingly, clinical management should be guided by the underlying pathophysiological mechanisms and implemented through targeted nurse-led and multidisciplinary interventions. These interventions may be applied in a stepwise manner, ranging from nutritional optimization to pharmacological glycemic control, with adjustments based on real-time metabolic responses. The following sections outline key intervention domains, including nutritional strategies, glucose monitoring, insulin therapy, and multidisciplinary coordination.

#### 3.3.1 Optimizing nutritional strategies

##### 3.3.1.1 Evidence-based selection of formula composition

Tailoring formula composition to a patient’s metabolic profile is a foundational step in mitigating EN-related hyperglycemia. Current ASPEN guidance emphasizes selecting diabetes-specific formulas (DSFs) for individuals with diabetes or those at elevated glycemic risk (23). These formulas typically combine slowly digestible carbohydrates with soluble fiber, thereby moderating intestinal glucose absorption and blunting postprandial excursions. Partial replacement of carbohydrates with monounsaturated fats or low–glycemic index carbohydrates is associated with improved insulin sensitivity and reduced exogenous insulin requirements (24). Clinical studies suggest that, compared with standard formulas, DSFs can reduce mean glucose concentrations by approximately 1–2 mmol/L and reduce insulin use by 15–25% in critically ill patients (25–27).

Beyond macronutrient adjustments, modifying fatty acid profiles, such as optimizing *ω*-6 to *ω*-3 ratios or using specialized lipid emulsions, may attenuate inflammation and contribute indirectly to improved glycemic stability (28). In routine practice, nurses play a critical role in monitoring tolerance following formula changes, documenting patterns of glycemia, gastrointestinal symptoms, and patient responses. Persistent hyperglycemia above 10 mmol/L within the first 48 h of EN initiation should prompt reassessment of formula choice, consideration of peptide-based alternatives, or early adjustment of insulin therapy to prevent further metabolic deterioration.

##### 3.3.1.2 Infusion kinetics and blood glucose homeostasis

Adjusting the rate and method of EN delivery offers another opportunity to stabilize glucose levels. Initiating EN at full caloric targets may overwhelm metabolic capacity and exacerbate hyperglycemia, particularly in the early phases of critical illness. Gradual advancement, beginning with 60–70% of caloric goals, helps minimize glycemic fluctuations and has been associated with improved outcomes (1, 29). Nurses ensure precise delivery through infusion pumps and closely document tolerance during titration.

The method of infusion also influences glycemic patterns. Continuous feeding generally produces smoother glucose trajectories and is often preferred for patients with significant metabolic instability (30). In contrast, bolus or intermittent feeding can provoke abrupt peaks and may increase aspiration risk among ventilated or elderly patients (31). Nevertheless, some individuals may tolerate intermittent delivery once their clinical status stabilizes, as it more closely mimics physiological eating rhythms. Volume-based feeding protocols have additionally demonstrated improved nutrient delivery and reduced glycemic variability by enabling flexible adjustments based on missed feeding time (32, 33).

An intimate relationship exists between hyperglycemia and feeding intolerance: elevated glucose impairs gastric motility, increases residual volumes, and predisposes to interruptions in EN delivery (34). Sustained glucose levels above 11.1 mmol/L may significantly heighten intolerance risk, underscoring the importance of timely insulin adjustment and thorough bedside monitoring (35).

#### 3.3.2 Glycemic monitoring and insulin therapy

Effective glucose monitoring forms the backbone of glycemic management in EN-fed critically ill patients. Arterial or venous sampling remains the most reliable method for obtaining glucose measurements, particularly when perfusion is compromised (36). Continuous glucose monitoring (CGM) has emerged as a valuable adjunct by offering real-time trends and reducing the frequency of undetected excursions. In several studies, CGM has been shown to improve time in range and reduce hypoglycemic episodes, enabling nurses to adjust insulin therapy more proactively. Automated insulin titration algorithms, when paired with CGM, have shown promise in further enhancing safety and stability.

Current SCCM recommendations suggest initiating structured glycemic management for enteral nutrition patients with persistent hyperglycemia, defined as two consecutive readings at or exceeding 10 mmol/L (37). Continuous intravenous insulin remains the preferred method in unstable patients because of its rapid adjustability, whereas basal–bolus regimens may be suitable once clinical status stabilizes. Standardized infusion protocols augmented by decision-support tools enhance safety, efficacy, and efficiency in clinical practice (38–40). Transitioning from intravenous to subcutaneous insulin requires careful recalibration, typically converting to 70–80% of the previous intravenous dose, to prevent rebound hyperglycemia (27, 41).

Hypoglycemia remains an important complication, particularly when feeding is interrupted, metabolic reserves are depleted, or renal and hepatic dysfunction alter insulin kinetics (42). Management follows a tiered response: providing carbohydrate supplementation for mild events, administering intravenous dextrose for symptomatic episodes, and reserving glucagon for severe hypoglycemia. Nurses’ continuous documentation of glucose patterns and interventions is essential for refining insulin protocols and preventing recurrent episodes (43).

#### 3.3.3 Management considerations for special patient groups

##### 3.3.3.1 Neurocritical care patients

Hyperglycemia is particularly detrimental for patients with neurological injury, where impaired cerebral glucose regulation may worsen ischemic damage and hinder neurological recovery. Moderated glycemic targets, typically 6.1–7.8 mmol/L for non-diabetic patients and slightly higher ranges for those with diabetes, are recommended to avoid both hyperglycemia and glucose deprivation. Warmed continuous infusions can help reduce feeding intolerance and stabilize glycemic fluctuations (44). Nurses monitor neurological status alongside glucose trends and adjust feeding rates or insulin therapy promptly when glucose exceeds 10 mmol/L.

##### 3.3.3.2 Patients with diabetes

For patients with established diabetes, individualized targets generally fall between 6 and 12 mmol/L (45, 46). Those with type 1 diabetes require uninterrupted basal insulin coverage even when feeding is paused, whereas individuals with type 2 diabetes often benefit from basal–bolus strategies (47). Diabetes-specific formulas rich in monounsaturated fats or low-glycemic carbohydrates may help reduce variability (48). Nurses provide essential oversight by documenting glycemic responses during feeding pauses and ensuring that insulin adjustments do not precipitate hypoglycemia.

##### 3.3.3.3 Postoperative surgical patients

Surgical stress markedly elevates glucose levels, and postoperative patients are particularly vulnerable to hyperglycemia-related complications. Evidence suggests that DSFs may improve insulin sensitivity after major surgery (49), while in cardiac surgery patients, coordinated insulin protocols and appropriately timed intermittent feeding improve glycemic control and ventilation outcomes (50). In patients undergoing abdominal or bowel surgery, particularly those with bowel resection, intestinal edema, impaired absorption, and altered gastrointestinal motility may contribute to increased glycemic variability and stress-induced insulin resistance (51). In this subgroup, glycemic management should be individualized with close glucose monitoring, early insulin initiation when indicated, and regular assessment of wound healing, blood glucose levels, and EN tolerance to enable timely nursing adjustments in caloric density or formula composition. Continuous or slow-intermittent feeding, cautious advancement of nutrition, and appropriate formula selection may further help reduce glycemic excursions while maintaining nutritional adequacy.

#### 3.3.4 Multidisciplinary collaboration

Optimal glycemic control during EN requires coordinated input from multidisciplinary teams. Collaboration among intensivists, endocrinologists, dietitians, pharmacists, and nursing staff supports the development of individualized insulin protocols and ensures that nutritional strategies align with metabolic status. Diabetes management teams have demonstrated benefits in improving glycemic stability, reducing infection rates, and facilitating smoother transitions from tube feeding to oral intake (52). Nutrition support teams similarly play a pivotal role in implementing standardized feeding protocols, guiding formula selection, and troubleshooting feeding intolerance (53). Across studies, structured team-based approaches consistently enhance time in range, reduce hypoglycemia, and improve adherence to best-practice guidelines.

**Figure 3:**
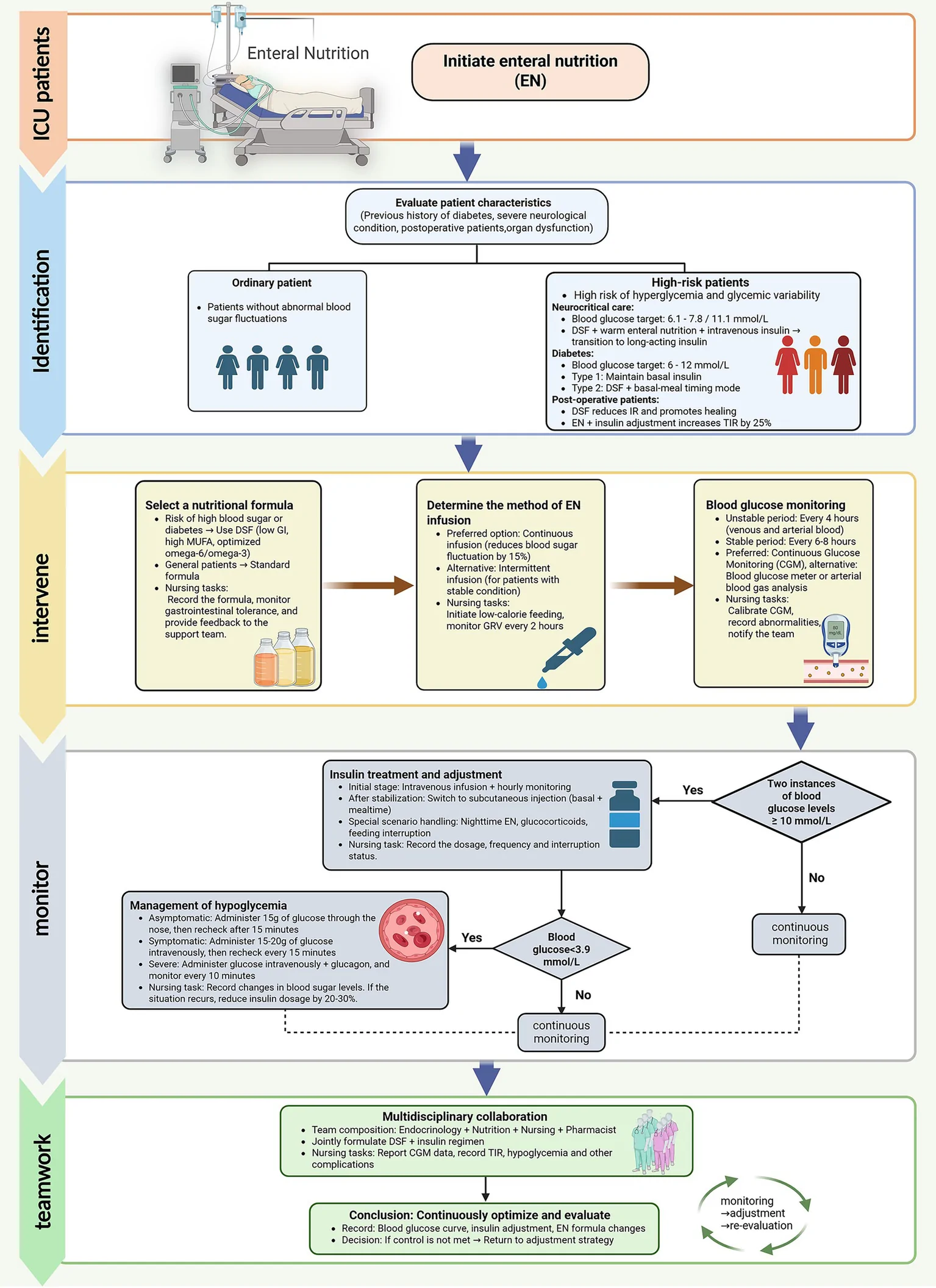
Flowchart of nursing-led glycemic management during EN in critically ill patients. The flowchart outlines an evidence-based nursing strategy for optimizing glycemic control in critically ill patients receiving enteral nutrition (EN) in the ICU. It integrates risk stratification, targeted nutrition, glucose monitoring, and adaptive insulin therapy into a nurse-coordinated feedback loop. Patients are classified as standard- or high-risk based on baseline metabolic status, guiding subsequent interventions. Interventions include formula selection, feeding modality, and glucose monitoring. Defined thresholds for hyperglycemia (≥10 mmol/L) and hypoglycemia (≤3.9 mmol/L) trigger insulin adjustment or correction. Insulin therapy is continuously tailored to clinical condition, feeding pattern, and glycemic variability. Nurses coordinate all stages, including monitoring, implementation, documentation, and multidisciplinary communication. EN, enteral nutrition; CGM, continuous glucose monitoring; GRV, gastric residual volume; TIR, time in range; DSF, diabetes-specific formula.

From a nursing perspective, the cascade model provides a structured framework to guide bedside assessment and intervention. Nurses play a central role in coordinating feeding practices, monitoring glycemic trends, identifying early deviations, and adjusting care plans in collaboration with multidisciplinary teams. This model may facilitate more proactive and individualized nursing decision-making, particularly in dynamic ICU environments (see Figure 3).

## 4 Discussion

This review focuses on enteral nutrition–associated hyperglycemia in critically ill patients as a clinically relevant condition during intensive care nutrition support and summarizes its multifactorial nature. Rather than introducing new biological mechanisms, it provides an integrated perspective on metabolic changes related to enteral feeding. Previous studies have mainly addressed this topic from single perspectives, including nutritional interventions, glucose-lowering strategies, or isolated therapeutic approaches, limiting comprehensive understanding. We propose a unified conceptual framework integrating epidemiological and clinical evidence to describe the sequential components of enteral nutrition–related hyperglycemia. This framework may support earlier identification of at-risk patients and more precise glucose monitoring and individualized adjustment of nutrition and insulin therapy. Effective management requires coordinated multidisciplinary nursing practice in intensive care, integrating enteral feeding, glucose monitoring, and insulin therapy.

However, current evidence remains heterogeneous. Continuous enteral feeding has been associated with more stable glycemic profiles in some studies, although other studies have reported no significant advantage over intermittent feeding (30). In contrast, diabetes-specific formulas and macronutrient modulation show relatively more consistent evidence of benefit, particularly in reducing glycemic variability and insulin requirements (25, 48, 54–56). Emerging approaches such as incretin-based therapies and microbiota-targeted interventions are supported mainly by early-stage and limited clinical evidence (18, 57, 58). Overall, these inconsistencies suggest that glycemic control in this setting is highly context-dependent and reflects dynamic interactions among nutrition delivery, host metabolic status, and gut-related factors.

From a clinical implementation perspective, effective glycemic management relies on continuous monitoring and structured workflow integration. Continuous glucose monitoring (CGM) has emerged as a valuable adjunct by providing real-time glucose trends and improving the detection of glycemic excursions that may be missed by intermittent sampling. CGM may facilitate more timely recognition of glucose fluctuations and support proactive insulin adjustment in critically ill patients receiving enteral nutrition (59). Automated insulin titration algorithms and nurse-managed insulin protocols may further enhance glycemic safety and stability (60, 61). Although current guidelines recommend initiating insulin therapy at approximately 10 mmol/L (62, 63), the optimal glycemic target remains uncertain. Advanced technologies such as automated insulin delivery approaches are promising but require further validation in critically ill populations (64).

Future research should focus on improving the understanding of enteral nutrition–related hyperglycemia in critically ill patients, particularly through prospective studies evaluating individualized nutritional strategies, glucose monitoring approaches, and insulin management protocols across different patient subgroups. Further investigation is also needed to clarify the roles of incretin signaling, gut microbiota modulation, and continuous glucose monitoring in optimizing glycemic control. Although the proposed cascade framework may serve as a useful conceptual tool for organizing current evidence, future efforts should primarily focus on generating high-quality clinical evidence to support precision glycemic management and improve patient outcomes.

### 4.1 Implications for practice

These findings apply mainly to critically ill adults in the ICU receiving enteral nutrition, especially those with metabolic vulnerability or persistent hyperglycemia. In clinical practice, this framework may guide stage-based management by linking mechanisms with targeted interventions, including early risk stratification, individualized formula selection, feeding adjustment, and timely insulin titration, which are indicated when blood glucose persistently exceeds 10 mmol/L on two consecutive measurements or when glycemic variability increases during feeding. Given the dynamic nature of glycemic status in critically ill patients, nurses play a central role in real-time glucose monitoring, early detection of instability, and coordination within multidisciplinary teams, supporting timely and individualized glycemic management in ICU settings.

### 4.2 Limitations

This review has several limitations that should be acknowledged. First, evidence for emerging interventions such as gut microbiota modulation and DPP-4 inhibitors is preliminary and hypothesis-generating, lacking robust multicenter validation. Second, substantial heterogeneity in insulin regimens, nutritional formulas and monitoring practices across included studies impairs the generalizability of our findings. Third, the proposed six-level cascade model remains a conceptual framework that requires prospective validation before routine clinical application. Additionally, few studies examine genetic, disease-specific differences in glycemic responses to enteral nutrition, and long-term outcomes beyond the acute ICU phase remain poorly characterized.

## 5 Conclusion

This review proposes a six-level cascade conceptual model to integrate current understanding of enteral nutrition–induced hyperglycemia in critical illness. While the model provides a structured and clinically relevant framework, it remains theoretical and requires validation. Nursing-led, individualized glycemic management strategies can improve care delivery, although further high-quality studies are needed to confirm their effectiveness.

## Data Availability

The original contributions presented in the study are included in the article/Supplementary material, further inquiries can be directed to the corresponding authors.
