# Correction: Enteral nutrition–related hyperglycemia in critical illness: cascade conceptual model and implications for precision nursing—a systematic review

**DOI:** 10.3389/fnut.2026.2000172

**Published:** 2026-09-29

**Authors:** Jiani Qian, Quanquan He, Maoyun Miao, Weiwei Ni, Yulei Ma

**Affiliations:** 1 School of Nursing, Shandong Second Medical University, Weifang, China; 2 Department of Nursing, Affiliated Hospital of Shandong Second Medical University, Weifang, China; 3 Weifang Key Laboratory for Clinical Nursing Research, Department of Nursing, Affiliated Hospital of Shandong Second Medical University, Weifang, China

**Keywords:** blood glucose, critical illness, enteral nutrition, glucose monitoring, continuous, hyperglycemia, insulin, nursing care

Authors Jiani Qian and Quanquan He were erroneously omitted as equal contributing authors.

The original version of this article has been updated.

