# Supplementary material for: Enteral nutrition–related hyperglycemia in critical illness: cascade conceptual model and implications for precision nursing—a systematic review
Source: Front Nutr. 2026 Jul 7;13:1803501. doi: 10.3389/fnut.2026.1803501 (PMC13385416; doi:10.3389/fnut.2026.1803501)
Supplement: Supplementary file 1 [file Table_1.DOCX]

# **1.Search strategy**

| Databases | Serial number | Search term | Literature quantity |
| --- | --- | --- | --- |
| pubmed | 1 | ​"Enteral nutrition"AND"Blood glucose" | 1066 |
|  | 2 | ​"Enteral nutrition"AND"Glucose levels" | 642 |
|  | 3 | ​"Enteral nutrition"AND"Hyperglycemia" | 500 |
|  | 4 | ​"Enteral nutrition"AND"​Hypoglycemia" | 296 |
|  | 5 | ​"Enteral nutrition"AND"Glycemic control" | 258 |
|  | 6 | ​"Enteral nutrition"AND"Glucose management" | 322 |
|  | 7 | ​"Enteral nutrition"AND"Blood glucose monitoring" | 145 |
|  | 8 | ​"Enteral nutrition"AND"Glycemic variability " | 60 |
|  | 9 | ​"Enteral nutrition"AND"Diabetes mellitus" | 424 |
|  | 10 | ​"Enteral nutrition"AND"​Insulin resistance" | 147 |
|  | 11 | ​"Enteral nutrition"AND"Gestational diabetes" | 19 |
|  | 12 | ​"Enteral nutrition"AND"​Insulin therapy" | 856 |
|  | 13 | ​"Enteral nutrition"AND"Postprandial blood glucose" | 72 |
|  | 14 | ​"Enteral nutrition"AND"stress hyperglycemia" | 77 |
|  | 15 | "gastric tube feeding"AND"Blood glucose" | 1090 |
|  | 16 | "gastric tube feeding"AND"Glucose levels" | 659 |
|  | 17 | "gastric tube feeding"AND"Hyperglycemia" | 505 |
|  | 18 | "gastric tube feeding"AND"​Hypoglycemia" | 307 |
|  | 19 | "gastric tube feeding"AND"Glycemic control" | 218 |
|  | 20 | "gastric tube feeding"AND"Glucose management" | 326 |
|  | 21 | "gastric tube feeding"AND"Blood glucose monitoring" | 148 |
|  | 22 | "gastric tube feeding"AND"Glycemic variability " | 60 |
|  | 23 | "gastric tube feeding"AND"Diabetes mellitus" | 438 |
|  | 24 | "gastric tube feeding"AND"​Insulin resistance" | 151 |
|  | 25 | "gastric tube feeding"AND"Gestational diabetes" | 19 |
|  | 26 | "gastric tube feeding"AND"Insulin therapy" | 870 |
|  | 27 | "gastric tube feeding"AND"Postprandial blood glucose" | 77 |
|  | 28 | "gastric tube feeding"AND"stress hyperglycemia" | 78 |
|  | 29 | "tube feeding"AND"Blood glucose" | 1181 |
|  | 30 | "tube feeding"AND"Glucose levels" | 728 |
|  | 31 | "tube feeding"AND"Hyperglycemia" | 523 |
|  | 32 | "tube feeding"AND"​Hypoglycemia" | 361 |
|  | 33 | "tube feeding"AND"Glycemic control" | 228 |
|  | 34 | "tube feeding"AND"Glucose management" | 362 |
|  | 35 | "tube feeding"AND"Blood glucose monitoring" | 165 |
|  | 36 | "tube feeding"AND"Glycemic variability " | 60 |
|  | 37 | "tube feeding"AND"Diabetes mellitus" | 506 |
|  | 38 | "tube feeding"AND"​Insulin resistance" | 161 |
|  | 39 | "tube feeding"AND"Gestational diabetes" | 31 |
|  | 40 | "tube feeding"AND"Insulin therapy" | 909 |
|  | 41 | "tube feeding"AND"Postprandial blood glucose" | 86 |
|  | 42 | "tube feeding"AND"stress hyperglycemia" | 82 |
|  | 43 | "enteral feeding"AND"Blood glucose" | 1132 |
|  | 44 | "enteral feeding"AND"Glucose levels" | 684 |
|  | 45 | "enteral feeding"AND"Hyperglycemia" | 521 |
|  | 46 | "enteral feeding"AND"​Hypoglycemia" | 338 |
|  | 47 | "enteral feeding"AND"Glycemic control" | 223 |
|  | 48 | "enteral feeding"AND"Glucose management" | 346 |
|  | 49 | "enteral feeding"AND"Blood glucose monitoring" | 156 |
|  | 50 | "enteral feeding"AND"Glycemic variability " | 61 |
|  | 51 | "enteral feeding"AND"Diabetes mellitus" | 459 |
|  | 52 | "enteral feeding"AND"​Insulin resistance" | 155 |
|  | 53 | "enteral feeding"AND"Gestational diabetes" | 25 |
|  | 54 | "enteral feeding"AND"Insulin therapy" | 898 |
|  | 55 | "enteral feeding"AND"Postprandial blood glucose" | 79 |
|  | 56 | "enteral feeding"AND"stress hyperglycemia" | 80 |
|  | 57 | "Gastrointestinal Feeding"AND"Blood glucose" | 222 |
|  | 58 | "astrointestinal feeding"AND"Glucose levels" | 107 |
|  | 59 | "astrointestinal feeding"AND"Hyperglycemia" | 88 |
|  | 60 | "astrointestinal feeding"AND"Glycemic control" | 39 |
|  | 61 | "astrointestinal feeding"AND"Glycemic variability " | 2 |
|  | 62 | "Intragastric Feeding"AND"Blood glucose" | 49 |
|  | 63 | "Intragastric Feeding"AND"Glycemic control" | 1 |
|  | 64 | "gastrostomy nutrition" AND "Glycemic control" | 12 |
|  | 65 | "gastrostomy nutrition"AND "Hyperglycemia" | 16 |
|  | 66 | "jejunostomy nutrition" AND"Blood glucose" | 15 |
|  | 67 | "jejunostomy nutrition" AND"Hyperglycemia" | 12 |
|  | 68 | "jejunostomy nutrition" AND"Glycemic control" | 10 |
|  | 69 | "enteral feed"AND "Hyperglycemia" | 504 |
|  | 70 | "enteral feed"AND "Blood glucose" | 372 |
|  | 71 | "enteral feed"AND "Glycemic control" | 176 |
|  | 72 | "enteral feed"AND "Glucose management" | 22 |
|  | 73 | "nasogastric feed*"AND"Blood glucose" | 31 |
|  | 74 | "nasogastric feed*"AND"Hyperglycemia" | 21 |
|  | total |  | 22069 |
| wos | 1 | ​"Enteral nutrition"AND"Blood glucose" | 344 |
|  | 2 | ​"Enteral nutrition"AND"Glucose levels" | 272 |
|  | 3 | ​"Enteral nutrition"AND"Hyperglycemia" | 343 |
|  | 4 | ​"Enteral nutrition"AND"​Hypoglycemia" | 147 |
|  | 5 | ​"Enteral nutrition"AND"Glycemic control" | 225 |
|  | 6 | ​"Enteral nutrition"AND"Glucose management" | 208 |
|  | 7 | ​"Enteral nutrition"AND"Blood glucose monitoring" | 66 |
|  | 8 | ​"Enteral nutrition"AND"Glycemic variability " | 50 |
|  | 9 | ​"Enteral nutrition"AND"Diabetes mellitus" | 191 |
|  | 10 | ​"Enteral nutrition"AND"​Insulin resistance" | 200 |
|  | 11 | ​"Enteral nutrition"AND"Gestational diabetes" | 22 |
|  | 12 | ​"Enteral nutrition"AND"​Insulin therapy" | 322 |
|  | 13 | ​"Enteral nutrition"AND"Postprandial blood glucose" | 37 |
|  | 14 | ​"Enteral nutrition"AND"stress hyperglycemia" | 69 |
|  | 15 | "gastric tube feeding"AND"Blood glucose" | 22 |
|  | 16 | "gastric tube feeding"AND"Glucose levels" | 20 |
|  | 17 | "gastric tube feeding"AND"Hyperglycemia" | 12 |
|  | 18 | "gastric tube feeding"AND"​Hypoglycemia" | 11 |
|  | 19 | "gastric tube feeding"AND"Glycemic control" | 8 |
|  | 20 | "gastric tube feeding"AND"Glucose management" | 10 |
|  | 21 | "gastric tube feeding"AND"Blood glucose monitoring" | 5 |
|  | 22 | "gastric tube feeding"AND"Glycemic variability " | 1 |
|  | 23 | "gastric tube feeding"AND"Diabetes mellitus" | 10 |
|  | 24 | "gastric tube feeding"AND"​Insulin resistance" | 13 |
|  | 25 | "gastric tube feeding"AND"Insulin therapy" | 11 |
|  | 26 | "tube feeding"AND"Blood glucose" | 119 |
|  | 27 | "tube feeding"AND"Glucose levels" | 124 |
|  | 28 | "tube feeding"AND"Hyperglycemia" | 61 |
|  | 29 | "tube feeding"AND"​Hypoglycemia" | 61 |
|  | 30 | "tube feeding"AND"Glycemic control" | 48 |
|  | 31 | "tube feeding"AND"Glucose management" | 53 |
|  | 32 | "tube feeding"AND"Blood glucose monitoring" | 17 |
|  | 33 | "tube feeding"AND"Glycemic variability " | 11 |
|  | 34 | "tube feeding"AND"Diabetes mellitus" | 92 |
|  | 35 | "tube feeding"AND"​Insulin resistance" | 44 |
|  | 36 | "tube feeding"AND"Insulin therapy" | 40 |
|  | 37 | "tube feeding"AND"Postprandial blood glucose" | 9 |
|  | 38 | "enteral feeding"AND"Blood glucose" | 153 |
|  | 39 | "enteral feeding"AND"Glucose levels" | 122 |
|  | 40 | "enteral feeding"AND"Hyperglycemia" | 122 |
|  | 41 | "enteral feeding"AND"​Hypoglycemia" | 88 |
|  | 42 | "enteral feeding"AND"Glycemic control" | 80 |
|  | 43 | "enteral feeding"AND"Glucose management" | 87 |
|  | 44 | "enteral feeding"AND"Blood glucose monitoring" | 26 |
|  | 45 | "enteral feeding"AND"Glycemic variability " | 21 |
|  | 46 | "enteral feeding"AND"Diabetes mellitus" | 74 |
|  | 47 | "enteral feeding"AND"​Insulin resistance" | 80 |
|  | 48 | "enteral feeding"AND"Insulin therapy" | 114 |
|  | 49 | "enteral feeding"AND"Postprandial blood glucose" | 15 |
|  | 50 | "enteral feeding"AND"stress hyperglycemia" | 23 |
|  | 51 | "Gastrointestinal Feeding"AND"Blood glucose" | 391 |
|  | 52 | "astrointestinal feeding"AND"Hyperglycemia" | 80 |
|  | 53 | "astrointestinal feeding"AND"Glycemic control" | 71 |
|  | 54 | "gastrostomy nutrition" AND "Glycemic control" | 11 |
|  | 55 | "enteral feed"AND "Hyperglycemia" | 118 |
|  | 56 | "enteral feed"AND "Blood glucose" | 148 |
|  | 57 | "enteral feed"AND "Glycemic control" | 74 |
|  | 58 | "enteral feed"AND "Glucose management" | 83 |
|  | 59 | "nasogastric feed*"AND"Blood glucose" | 20 |
|  | 60 | "nasogastric feed*"AND"Hyperglycemia" | 15 |
|  | total |  | 5314 |
| embase | 1 | ​"Enteral nutrition"AND"Blood glucose" | 943 |
|  | 2 | ​"Enteral nutrition"AND"Glucose levels" | 394 |
|  | 3 | ​"Enteral nutrition"AND"Hyperglycemia" | 833 |
|  | 4 | ​"Enteral nutrition"AND"​Hypoglycemia" | 618 |
|  | 5 | ​"Enteral nutrition"AND"Glycemic control" | 406 |
|  | 6 | ​"Enteral nutrition"AND"Glucose management" | 54 |
|  | 7 | ​"Enteral nutrition"AND"Blood glucose monitoring" | 43 |
|  | 8 | ​"Enteral nutrition"AND"Glycemic variability " | 43 |
|  | 9 | ​"Enteral nutrition"AND"Diabetes mellitus" | 690 |
|  | 10 | ​"Enteral nutrition"AND"​Insulin resistance" | 398 |
|  | 11 | ​"Enteral nutrition"AND"Gestational diabetes" | 26 |
|  | 12 | ​"Enteral nutrition"AND"​Insulin therapy" | 226 |
|  | 13 | ​"Enteral nutrition"AND"Postprandial blood glucose" | 27 |
|  | 14 | ​"Enteral nutrition"AND"stress hyperglycemia" | 36 |
|  | 15 | "gastric tube feeding"AND"Blood glucose" | 69 |
|  | 16 | "gastric tube feeding"AND"Glucose levels" | 24 |
|  | 17 | "gastric tube feeding"AND"Hyperglycemia" | 39 |
|  | 18 | "gastric tube feeding"AND"​Hypoglycemia" | 60 |
|  | 19 | "gastric tube feeding"AND"Glycemic control" | 26 |
|  | 20 | "gastric tube feeding"AND"Glucose management" | 2 |
|  | 21 | "gastric tube feeding"AND"Blood glucose monitoring" | 2 |
|  | 22 | "gastric tube feeding"AND"Glycemic variability " | 3 |
|  | 23 | "gastric tube feeding"AND"Diabetes mellitus" | 86 |
|  | 24 | "gastric tube feeding"AND"​Insulin resistance" | 17 |
|  | 25 | "gastric tube feeding"AND"Insulin therapy" | 5 |
|  | 26 | "gastric tube feeding"AND"Postprandial blood glucose" | 5 |
|  | 27 | "tube feeding"AND"Blood glucose" | 298 |
|  | 28 | "tube feeding"AND"Glucose levels" | 131 |
|  | 29 | "tube feeding"AND"Hyperglycemia" | 220 |
|  | 30 | "tube feeding"AND"​Hypoglycemia" | 289 |
|  | 31 | "tube feeding"AND"Glycemic control" | 92 |
|  | 32 | "tube feeding"AND"Glucose management" | 16 |
|  | 33 | "tube feeding"AND"Blood glucose monitoring" | 11 |
|  | 34 | "tube feeding"AND"Glycemic variability " | 11 |
|  | 35 | "tube feeding"AND"Diabetes mellitus" | 356 |
|  | 36 | "tube feeding"AND"​Insulin resistance" | 75 |
|  | 37 | "tube feeding"AND"Gestational diabetes" | 29 |
|  | 38 | "tube feeding"AND"Insulin therapy" | 37 |
|  | 39 | "tube feeding"AND"Postprandial blood glucose" | 11 |
|  | 40 | "tube feeding"AND"stress hyperglycemia" | 3 |
|  | 41 | "enteral feeding"AND"Blood glucose" | 825 |
|  | 42 | "enteral feeding"AND"Glucose levels" | 349 |
|  | 43 | "enteral feeding"AND"Hyperglycemia" | 725 |
|  | 44 | "enteral feeding"AND"​Hypoglycemia" | 569 |
|  | 45 | "enteral feeding"AND"Glycemic control" | 333 |
|  | 46 | "enteral feeding"AND"Glucose management" | 42 |
|  | 47 | "enteral feeding"AND"Blood glucose monitoring" | 34 |
|  | 48 | "enteral feeding"AND"Glycemic variability " | 41 |
|  | 49 | "enteral feeding"AND"Diabetes mellitus" | 557 |
|  | 50 | "enteral feeding"AND"​Insulin resistance" | 276 |
|  | 51 | "enteral feeding"AND"Gestational diabetes" | 23 |
|  | 52 | "enteral feeding"AND"Insulin therapy" | 202 |
|  | 53 | "enteral feeding"AND"Postprandial blood glucose" | 19 |
|  | 54 | "enteral feeding"AND"stress hyperglycemia" | 29 |
|  | 55 | "Gastrointestinal Feeding"AND"Blood glucose" | 408 |
|  | 56 | "astrointestinal feeding"AND"Glucose levels" | 168 |
|  | 57 | "astrointestinal feeding"AND"Hyperglycemia" | 230 |
|  | 58 | "astrointestinal feeding"AND"Glycemic control" | 107 |
|  | 59 | "astrointestinal feeding"AND"Glycemic variability " | 9 |
|  | 60 | "Intragastric Feeding"AND"Blood glucose" | 803 |
|  | 61 | "Intragastric Feeding"AND"Glycemic control" | 289 |
|  | 62 | "Intragastric Feeding"AND"Hyperglycemia" | 606 |
|  | 63 | "Intragastric Feeding"AND"Glucose management" | 36 |
|  | 64 | "Intragastric Feeding"AND"Blood glucose monitoring" | 30 |
|  | 65 | "gastrostomy nutrition" AND "Glycemic control" | 32 |
|  | 66 | "gastrostomy nutrition"AND "Hyperglycemia" | 43 |
|  | 67 | "jejunostomy nutrition" AND"Blood glucose" | 31 |
|  | 68 | "jejunostomy nutrition" AND"Hyperglycemia" | 21 |
|  | 69 | "jejunostomy nutrition" AND"Glycemic control" | 21 |
|  | 70 | "enteral feed"AND "Hyperglycemia" | 24 |
|  | 71 | "enteral feed"AND "Blood glucose" | 53 |
|  | 72 | "enteral feed"AND "Glycemic control" | 13 |
|  | 73 | "nasogastric feed*"AND"Blood glucose" | 100 |
|  | 74 | "nasogastric feed*"AND"Hyperglycemia" | 52 |
|  | total |  | 13754 |
| cochrane | 1 | ​"Enteral nutrition"AND"Blood glucose" | 561 |
|  | 2 | ​"Enteral nutrition"AND"Glucose levels" | 430 |
|  | 3 | ​"Enteral nutrition"AND"Hyperglycemia" | 163 |
|  | 4 | ​"Enteral nutrition"AND"​Hypoglycemia" | 110 |
|  | 5 | ​"Enteral nutrition"AND"Glycemic control" | 167 |
|  | 6 | ​"Enteral nutrition"AND"Glucose management" | 130 |
|  | 7 | ​"Enteral nutrition"AND"Blood glucose monitoring" | 130 |
|  | 8 | ​"Enteral nutrition"AND"Glycemic variability " | 47 |
|  | 9 | ​"Enteral nutrition"AND"Diabetes mellitus" | 224 |
|  | 10 | ​"Enteral nutrition"AND"​Insulin resistance" | 85 |
|  | 11 | ​"Enteral nutrition"AND"Gestational diabetes" | 28 |
|  | 12 | ​"Enteral nutrition"AND"​Insulin therapy" | 216 |
|  | 13 | ​"Enteral nutrition"AND"Postprandial blood glucose" | 72 |
|  | 14 | ​"Enteral nutrition"AND"stress hyperglycemia" | 72 |
|  | 15 | "gastric tube feeding"AND"Blood glucose" | 60 |
|  | 16 | "gastric tube feeding"AND"Glucose levels" | 42 |
|  | 17 | "gastric tube feeding"AND"Hyperglycemia" | 15 |
|  | 18 | "gastric tube feeding"AND"​Hypoglycemia" | 7 |
|  | 19 | "gastric tube feeding"AND"Glycemic control" | 16 |
|  | 20 | "gastric tube feeding"AND"Glucose management" | 14 |
|  | 21 | "gastric tube feeding"AND"Blood glucose monitoring" | 15 |
|  | 22 | "gastric tube feeding"AND"Glycemic variability " | 2 |
|  | 23 | "gastric tube feeding"AND"Diabetes mellitus" | 19 |
|  | 24 | "gastric tube feeding"AND"​Insulin resistance" | 7 |
|  | 25 | "gastric tube feeding"AND"Insulin therapy" | 10 |
|  | 26 | "gastric tube feeding"AND"Postprandial blood glucose" | 15 |
|  | 27 | "tube feeding"AND"Blood glucose" | 193 |
|  | 28 | "tube feeding"AND"Glucose levels" | 142 |
|  | 29 | "tube feeding"AND"Hyperglycemia" | 56 |
|  | 30 | "tube feeding"AND"​Hypoglycemia" | 46 |
|  | 31 | "tube feeding"AND"Glycemic control" | 55 |
|  | 32 | "tube feeding"AND"Glucose management" | 46 |
|  | 33 | "tube feeding"AND"Blood glucose monitoring" | 54 |
|  | 34 | "tube feeding"AND"Glycemic variability " | 14 |
|  | 35 | "tube feeding"AND"Diabetes mellitus" | 73 |
|  | 36 | "tube feeding"AND"​Insulin resistance" | 15 |
|  | 37 | "tube feeding"AND"Gestational diabetes" | 12 |
|  | 38 | "tube feeding"AND"Insulin therapy" | 48 |
|  | 39 | "tube feeding"AND"Postprandial blood glucose" | 27 |
|  | 40 | "tube feeding"AND"stress hyperglycemia" | 8 |
|  | 41 | "enteral feeding"AND"Blood glucose" | 378 |
|  | 42 | "enteral feeding"AND"Glucose levels" | 300 |
|  | 43 | "enteral feeding"AND"Hyperglycemia" | 119 |
|  | 44 | "enteral feeding"AND"​Hypoglycemia" | 115 |
|  | 45 | "enteral feeding"AND"Glycemic control" | 100 |
|  | 46 | "enteral feeding"AND"Glucose management" | 80 |
|  | 47 | "enteral feeding"AND"Blood glucose monitoring" | 88 |
|  | 48 | "enteral feeding"AND"Glycemic variability " | 34 |
|  | 49 | "enteral feeding"AND"Diabetes mellitus" | 137 |
|  | 50 | "enteral feeding"AND"​Insulin resistance" | 48 |
|  | 51 | "enteral feeding"AND"Gestational diabetes" | 24 |
|  | 52 | "enteral feeding"AND"Insulin therapy" | 136 |
|  | 53 | "enteral feeding"AND"Postprandial blood glucose" | 45 |
|  | 54 | "enteral feeding"AND"stress hyperglycemia" | 20 |
|  | 55 | "Gastrointestinal Feeding"AND"Blood glucose" | 226 |
|  | 56 | "astrointestinal feeding"AND"Glucose levels" | 172 |
|  | 57 | "astrointestinal feeding"AND"Hyperglycemia" | 39 |
|  | 58 | "astrointestinal feeding"AND"Glycemic control" | 44 |
|  | 59 | "astrointestinal feeding"AND"Glycemic variability " | 16 |
|  | 60 | "Intragastric Feeding"AND"Blood glucose" | 23 |
|  | 61 | "Intragastric Feeding"AND"Glycemic control" | 4 |
|  | 62 | "Intragastric Feeding"AND"Hyperglycemia" | 3 |
|  | 63 | "Intragastric Feeding"AND"Glucose management" | 2 |
|  | 64 | "Intragastric Feeding"AND"Blood glucose monitoring" | 3 |
|  | 65 | "gastrostomy nutrition" AND "Glycemic control" | 100 |
|  | 66 | "gastrostomy nutrition"AND "Hyperglycemia" | 119 |
|  | 67 | "jejunostomy nutrition" AND"Blood glucose" | 12 |
|  | 68 | "jejunostomy nutrition" AND"Hyperglycemia" | 5 |
|  | 69 | "jejunostomy nutrition" AND"Glycemic control" | 3 |
|  | 70 | "enteral feed"AND "Hyperglycemia" | 119 |
|  | 71 | "enteral feed"AND "Blood glucose" | 378 |
|  | 72 | "enteral feed"AND "Glycemic control" | 100 |
|  | 73 | "enteral feed"AND "Glucose management" | 80 |
|  | 74 | "nasogastric feed*"AND"Blood glucose" | 73 |
|  | 75 | "nasogastric feed*"AND"Hyperglycemia" | 17 |
|  | total |  | 6608 |
| scopus | 1 | ​"Enteral nutrition"AND"Blood glucose" | 1419 |
|  | 2 | ​"Enteral nutrition"AND"Glucose levels" | 1359 |
|  | 3 | ​"Enteral nutrition"AND"Hyperglycemia" | 865 |
|  | 4 | ​"Enteral nutrition"AND"​Hypoglycemia" | 476 |
|  | 5 | ​"Enteral nutrition"AND"Glycemic control" | 422 |
|  | 6 | ​"Enteral nutrition"AND"Glucose management" | 396 |
|  | 7 | ​"Enteral nutrition"AND"Blood glucose monitoring" | 265 |
|  | 8 | ​"Enteral nutrition"AND"Glycemic variability " | 54 |
|  | 9 | ​"Enteral nutrition"AND"Diabetes mellitus" | 850 |
|  | 10 | ​"Enteral nutrition"AND"​Insulin resistance" | 256 |
|  | 11 | ​"Enteral nutrition"AND"Gestational diabetes" | 55 |
|  | 12 | ​"Enteral nutrition"AND"​Insulin therapy" | 560 |
|  | 13 | ​"Enteral nutrition"AND"Postprandial blood glucose" | 93 |
|  | 14 | ​"Enteral nutrition"AND"stress hyperglycemia" | 144 |
|  | 15 | "gastric tube feeding"AND"Blood glucose" | 114 |
|  | 16 | "gastric tube feeding"AND"Glucose levels" | 109 |
|  | 17 | "gastric tube feeding"AND"Hyperglycemia" | 42 |
|  | 18 | "gastric tube feeding"AND"​Hypoglycemia" | 37 |
|  | 19 | "gastric tube feeding"AND"Glycemic control" | 20 |
|  | 20 | "gastric tube feeding"AND"Glucose management" | 26 |
|  | 21 | "gastric tube feeding"AND"Blood glucose monitoring" | 22 |
|  | 22 | "gastric tube feeding"AND"Glycemic variability " | 1 |
|  | 23 | "gastric tube feeding"AND"Diabetes mellitus" | 71 |
|  | 24 | "gastric tube feeding"AND"​Insulin resistance" | 16 |
|  | 25 | "gastric tube feeding"AND"Insulin therapy" | 29 |
|  | 26 | "gastric tube feeding"AND"Postprandial blood glucose" | 22 |
|  | 27 | "tube feeding"AND"Blood glucose" | 582 |
|  | 28 | "tube feeding"AND"Glucose levels" | 548 |
|  | 29 | "tube feeding"AND"Hyperglycemia" | 251 |
|  | 30 | "tube feeding"AND"​Hypoglycemia" | 251 |
|  | 31 | "tube feeding"AND"Glycemic control" | 106 |
|  | 32 | "tube feeding"AND"Glucose management" | 148 |
|  | 33 | "tube feeding"AND"Blood glucose monitoring" | 102 |
|  | 34 | "tube feeding"AND"Glycemic variability " | 14 |
|  | 35 | "tube feeding"AND"Diabetes mellitus" | 513 |
|  | 36 | "tube feeding"AND"​Insulin resistance" | 84 |
|  | 37 | "tube feeding"AND"Gestational diabetes" | 42 |
|  | 38 | "tube feeding"AND"Insulin therapy" | 16 |
|  | 39 | "tube feeding"AND"Postprandial blood glucose" | 54 |
|  | 40 | "tube feeding"AND"stress hyperglycemia" | 26 |
|  | 41 | "enteral feeding"AND"Blood glucose" | 1241 |
|  | 42 | "enteral feeding"AND"Glucose levels" | 1184 |
|  | 43 | "enteral feeding"AND"Hyperglycemia" | 733 |
|  | 44 | "enteral feeding"AND"​Hypoglycemia" | 444 |
|  | 45 | "enteral feeding"AND"Glycemic control" | 358 |
|  | 46 | "enteral feeding"AND"Glucose management" | 344 |
|  | 47 | "enteral feeding"AND"Blood glucose monitoring" | 240 |
|  | 48 | "enteral feeding"AND"Glycemic variability " | 52 |
|  | 49 | "enteral feeding"AND"Diabetes mellitus" | 741 |
|  | 50 | "enteral feeding"AND"​Insulin resistance" | 192 |
|  | 51 | "enteral feeding"AND"Gestational diabetes" | 64 |
|  | 52 | "enteral feeding"AND"Insulin therapy" | 466 |
|  | 53 | "enteral feeding"AND"Postprandial blood glucose" | 95 |
|  | 54 | "enteral feeding"AND"stress hyperglycemia" | 121 |
|  | 55 | "Gastrointestinal Feeding"AND"Blood glucose" | 1360 |
|  | 56 | "Gastrointestinal feeding"AND"Glucose levels" | 1379 |
|  | 57 | "Gastrointestinal feeding"AND"Hyperglycemia" | 416 |
|  | 58 | "Gastrointestinal feeding"AND"Glycemic control" | 230 |
|  | 59 | "Gastrointestinal feeding"AND"Glycemic variability " | 17 |
|  | 60 | "Intragastric Feeding"AND"Blood glucose" | 171 |
|  | 61 | "Intragastric Feeding"AND"Glycemic control" | 3 |
|  | 62 | "Intragastric Feeding"AND"Hyperglycemia" | 17 |
|  | 63 | "Intragastric Feeding"AND"Glucose management" | 13 |
|  | 64 | "Intragastric Feeding"AND"Blood glucose monitoring" | 4 |
|  | 65 | "Intragastric Feeding"AND"Blood glucose" | 171 |
|  | 66 | "Intragastric Feeding"AND"Glycemic control" | 3 |
|  | 67 | "gastrostomy nutrition" AND "Glycemic control" | 25 |
|  | 68 | "gastrostomy nutrition"AND "Hyperglycemia" | 57 |
|  | 69 | "jejunostomy nutrition" AND"Blood glucose" | 57 |
|  | 70 | "jejunostomy nutrition" AND"Hyperglycemia" | 55 |
|  | 71 | "jejunostomy nutrition" AND"Glycemic control" | 23 |
|  | 72 | "enteral feed"AND "Hyperglycemia" | 76 |
|  | 73 | "enteral feed"AND "Blood glucose" | 148 |
|  | 74 | "enteral feed"AND "Glycemic control" | 44 |
|  | 75 | "enteral feed"AND "Glucose management" | 37 |
|  | 76 | "nasogastric feed*"AND"Blood glucose" | 153 |
|  | 77 | "nasogastric feed*"AND"Hyperglycemia" | 64 |
|  | total |  | 21258 |
| CINAHL | 1 | ​"Enteral nutrition"AND"Blood glucose" | 283 |
|  | 2 | ​"Enteral nutrition"AND"Glucose levels" | 85 |
|  | 3 | ​"Enteral nutrition"AND"Hyperglycemia" | 237 |
|  | 4 | ​"Enteral nutrition"AND"​Hypoglycemia" | 134 |
|  | 5 | ​"Enteral nutrition"AND"Glycemic control" | 185 |
|  | 6 | ​"Enteral nutrition"AND"Glucose management" | 21 |
|  | 7 | ​"Enteral nutrition"AND"Blood glucose monitoring" | 42 |
|  | 8 | ​"Enteral nutrition"AND"Glycemic variability " | 23 |
|  | 9 | ​"Enteral nutrition"AND"Diabetes mellitus" | 236 |
|  | 10 | ​"Enteral nutrition"AND"​Insulin resistance" | 54 |
|  | 11 | ​"Enteral nutrition"AND"Gestational diabetes" | 8 |
|  | 12 | ​"Enteral nutrition"AND"​Insulin therapy" | 39 |
|  | 13 | ​"Enteral nutrition"AND"Postprandial blood glucose" | 5 |
|  | 14 | ​"Enteral nutrition"AND"stress hyperglycemia" | 19 |
|  | 15 | "gastric tube feeding"AND"Blood glucose" | 3 |
|  | 16 | "gastric tube feeding"AND"Hyperglycemia" | 6 |
|  | 17 | "gastric tube feeding"AND"​Hypoglycemia" | 4 |
|  | 18 | "gastric tube feeding"AND"Glycemic variability " | 1 |
|  | 19 | "tube feeding"AND"Blood glucose" | 150 |
|  | 20 | "tube feeding"AND"Glucose levels" | 47 |
|  | 21 | "tube feeding"AND"Hyperglycemia" | 113 |
|  | 22 | "tube feeding"AND"​Hypoglycemia" | 69 |
|  | 23 | "tube feeding"AND"Glycemic control" | 99 |
|  | 24 | "tube feeding"AND"Glucose management" | 8 |
|  | 25 | "tube feeding"AND"Blood glucose monitoring" | 24 |
|  | 26 | "tube feeding"AND"Glycemic variability " | 14 |
|  | 27 | "tube feeding"AND"Diabetes mellitus" | 152 |
|  | 28 | "tube feeding"AND"​Insulin resistance" | 21 |
|  | 29 | "tube feeding"AND"Gestational diabetes" | 3 |
|  | 30 | "tube feeding"AND"Insulin therapy" | 16 |
|  | 31 | "tube feeding"AND"Postprandial blood glucose" | 4 |
|  | 32 | "tube feeding"AND"stress hyperglycemia" | 8 |
|  | 33 | "enteral feeding"AND"Blood glucose" | 171 |
|  | 34 | "enteral feeding"AND"Glucose levels" | 57 |
|  | 35 | "enteral feeding"AND"Hyperglycemia" | 135 |
|  | 36 | "enteral feeding"AND"​Hypoglycemia" | 86 |
|  | 37 | "enteral feeding"AND"Glycemic control" | 154 |
|  | 38 | "enteral feeding"AND"Glucose management" | 11 |
|  | 39 | "enteral feeding"AND"Blood glucose monitoring" | 38 |
|  | 40 | "enteral feeding"AND"Glycemic variability " | 14 |
|  | 41 | "enteral feeding"AND"Diabetes mellitus" | 144 |
|  | 42 | "enteral feeding"AND"​Insulin resistance" | 22 |
|  | 43 | "enteral feeding"AND"Gestational diabetes" | 4 |
|  | 44 | "enteral feeding"AND"Insulin therapy" | 23 |
|  | 45 | "enteral feeding"AND"Postprandial blood glucose" | 4 |
|  | 46 | "enteral feeding"AND"stress hyperglycemia" | 8 |
|  | 47 | "Gastrointestinal Feeding"AND"Blood glucose" | 3 |
|  | 48 | "astrointestinal feeding"AND"Glucose levels" | 2 |
|  | 49 | "astrointestinal feeding"AND"Hyperglycemia" | 2 |
|  | 50 | "Intragastric Feeding"AND"Blood glucose" | 3 |
|  | 51 | "jejunostomy nutrition" AND"Glycemic control" | 2 |
|  | 52 | "enteral feed"AND "Hyperglycemia" | 9 |
|  | 53 | "enteral feed"AND "Blood glucose" | 12 |
|  | 54 | "enteral feed"AND "Glycemic control" | 9 |
|  | 55 | "nasogastric feed*"AND"Blood glucose" | 12 |
|  | 56 | "nasogastric feed*"AND"Hyperglycemia" | 8 |
|  | total |  | 3046 |

# **2.search results**

| **Databases** | **Literature quantity** | **After removing duplicates** |
| --- | --- | --- |
| pubmed | 22069 | 2668 |
| wos | 5314 | 1228 |
| embase | 13754 | 2674 |
| cochrane | 6608 | 726 |
| scopus | 21258 | 3159 |
| cinahl | 3046 | 374 |
| total | 72049 | 10829 |

# **3.Summary table of studies included in the research**

| **Study** | **Design** | **Article Content** |
| --- | --- | --- |
| Yao, 2025 | Retrospective | Identified risk factors and supports individualized carbohydrate strategies |
| Honarmand, 2024 | Guideline | Recommends insulin infusion, frequent monitoring, and moderate targets |
| Hiemstra, 2024 | Retrospective | Persistent circadian glucose variability during continuous EN |
| Ni, 2024 | Retrospective | Intermediate/long-acting insulin effectively controls EN-related hyperglycemia |
| Rebollo-Pérez, 2023 | Expert consensus | Highlights lack of standardization and provides management guidance |
| Wu, 2023 | Expert consensus | Supports safe, individualized control minimizing hypoglycemia and variability |
| Seifi, 2022 | RCT | No metabolic benefit of synbiotic supplementation |
| Burslem, 2022 | Review | LCHF may benefit subgroups; evidence remains limited |
| Wang, 2021 | Retrospective | Minimal formula differences; low-carbohydrate may be advantageous |
| Ren, 2021 | RCT | Sequential feeding non-inferior; may reduce hyperglycemia |
| Masood, 2021 | Retrospective | Insulin protocols achieve targets safely |
| Seyyedi, 2020 | RCT | Feeding method affects phosphorus, not glycemia |
| Prest, 2020 | Retrospective | Volume-based feeding improves nutrition and glycemic outcomes |
| Murphy, 2020 | Review | Supports individualized targets with emerging technologies |
| Sarfo-Adu, 2019 | Retrospective | Requires early insulin adjustment and specialist involvement |
| Roberts, 2019 | Retrospective | Volume-based EN improves delivery without glycemic compromise |
| Doola, 2019 | RCT | Low-carbohydrate formulas reduce insulin requirements |
| Vahabzadeh, 2019 | RCT | High-fat formulas improve metabolism, not outcomes |
| Mader, 2019 | Prospective cohort | Decision-support systems are feasible and effective |
| Doola, 2019 | Retrospective | Glycemic variability predicts mortality risk |
| Steen, 2018 | RCT | Low-carbohydrate lowers mean glucose, not variability |
| Roberts, 2018 | Guideline | Recommends moderate targets and multidisciplinary care |
| Shao, 2018 | RCT | Diabetes-specific formulas improve acute glycemic control |

**Continued from the previous table**

| **Study** | **Design** | **Article Content** |
| --- | --- | --- |
| Egi, 2018 | RCT | Isomaltulose formulas improve control safely |
| Sanz-Paris, 2017 | Expert consensus | Supports individualized nutrition and metabolic targets |
| Drincic, 2017 | Expert consensus | Emphasizes synchronizing nutrition and insulin therapy |
| Xu, 2017 | Mixed-method | Hyperglycemia linked to feeding intolerance |
| McClave, 2016 | Guideline | EN preferred with individualized assessment |
| Mabrey, 2015 | Guideline | Advocates individualized insulin and nutrition strategies |
| Davidson, 2015 | Expert consensus | Supports moderate targets (140–180 mg/dL) |
| Wong, 2014 | Retrospective | Specialist teams improve glycemic outcomes |
| Murphy, 2014 | Retrospective | Intermittent feeding reduces hypoglycemia risk |
| Dickerson, 2014 | Review | Highlights importance of multidisciplinary teams |
| Amrein, 2014 | Prospective | SGC systems safe and effective |
| McMahon, 2013 | Guideline | Balances glycemic control and hypoglycemia risk |
| Gosmanov, 2013 | Expert consensus | Calls for optimized insulin and nutrition strategies |
| Gosmanov, 2013 | Review | Hyperglycemia linked to poor outcomes; optimize insulin |
| Yao, 2013 | RCT | Early EN improves insulin sensitivity vs PN |
| Shetty, 2012 | Prospective cohort | Validates safe insulin infusion protocols |
| Mori, 2012 | RCT | Low-carbohydrate formulas reduce variability |
| Ichai, 2010 | Guideline | Avoid severe hyperglycemia; use standardized insulin |
| Petrov, 2007 | Systematic review | EN improves control and reduces infection vs PN |
| Nguyen, 2007 | observational | Variability linked to feeding intolerance |
| Pachler, 2007 | RCT | eMPC improves glycemic control |
| Vriesendorp, 2006 | Retrospective | Identifies risk factors for ICU hypoglycemia |
